# Temporal Trends in the Swedish HIV-1 Epidemic: Increase in Non-B Subtypes and Recombinant Forms over Three Decades

**DOI:** 10.1371/journal.pone.0099390

**Published:** 2014-06-12

**Authors:** Ujjwal Neogi, Amanda Häggblom, Michele Santacatterina, Göran Bratt, Magnus Gisslén, Jan Albert, Anders Sonnerborg

**Affiliations:** 1 Division of Clinical Microbiology, Department of Laboratory Medicine, Karolinska Institutet, Stockholm, Sweden; 2 Unit of Infectious Diseases, Department of Medicine Huddinge, Karolinska Institutet, Stockholm, Sweden; 3 Department of Infectious Diseases, County Council of Gävleborg, Gävle, Sweden; 4 Unit of Biostatistics, Institute of Environmental Medicine, Karolinska Institutet, Stockholm, Sweden; 5 Department of Clinical Science and Education, Venhälsan, Stockholm South General Hospital, Stockholm, Sweden; 6 Department of Infectious Diseases, University of Gothenburg, Gothenburg, Sweden; 7 Department of Microbiology, Tumor and Cell Biology, Karolinska Institutet, Stockholm, Sweden; University of Athens, Medical School, Greece

## Abstract

**Background:**

HIV-1 subtype B (HIV-1B) still dominates in resource-rich countries but increased migration contributes to changes in the global subtype distribution. Also, spread of non-B subtypes within such countries occurs. The trend of the subtype distribution from the beginning of the epidemic in the country has earlier not been reported in detail. Thus the primary objective of this study is to describe the temporal trend of the subtype distribution from the beginning of the HIV-1 epidemic in Sweden over three decades.

**Methods:**

HIV-1 *pol* sequences from patients (n = 3967) diagnosed in Sweden 1983– 2012, corresponding to >40% of patients ever diagnosed, were re-subtyped using several automated bioinformatics tools. The temporal trends of subtypes and recombinants during three decades were described by a multinomial logistic regression model.

**Results:**

All eleven group M HIV-1 subtypes and sub-subtypes (78%), 17 circulating recombinant forms (CRFs) (19%) and 32 unique recombinants forms (URF) (3%) were identified. When all patients were analysed, there was an increase of newly diagnosed HIV-1C (RR, 95%CI: 1.10, 1.06–1.14), recombinants (1.20, 1.17–1.24) and other pure subtypes (1.11, 1.07–1.16) over time compared to HIV-1B. The same pattern was found when all patients infected in Sweden (n = 1165) were analysed. Also, for MSM patients infected in Sweden (n = 921), recombinant forms and other pure subtypes increased.

**Significance:**

Sweden exhibits one of the most diverse subtype epidemics outside Africa. The increase of non-B subtypes is due to migration and to a spread among heterosexually infected patients and MSM within the country. This viral heterogeneity may become a hotspot for development of more diverse and complex recombinant forms if the epidemics converge.

## Introduction

Significant disparity is observed in the global distribution of human immunodeficiency virus type 1 (HIV-1) subtypes [1]. The greatest viral diversity is found in west and central Africa, while the epidemic is largely mono-phylogenetic in other regions, e.g. South Africa, Ethiopia, and India where HIV-1 subtype C (HIV-1C) dominates [2]–[5]. In resource rich areas, the epidemic was driven during the first decades by HIV-1 subtype B (HIV-1B) [6]–[8], but an increase of non-B subtypes in Western Europe has been reported [9], [10]. However, cross-sectional data across 20 European countries show that HIV-1B is still predominant in all of the countries, ranging from 45% to 90% [7].

Representative information on the HIV-1 subtype distribution is valuable for tracking the epidemiology and understanding the evolutionary trajectory of the virus. With the advance of automated subtyping tools, the accuracy of subtype prediction and the feasibility to analyse large datasets have increased. However, subtyping of viral recombinant forms needs more stringent analysis and the use of atleast two of the bioinformatics tools available is recommended [11].

Sweden has a low prevalence (0.06%) and incidence of HIV-1 infection and the migration from high prevalence countries has therefore played an important role in the increased diversity of the subtypes since the 1990s [12]. Our previous study of patients diagnosed 1980–1993, showed that almost exclusively HIV-1B was circulating up to the end of the 1980s [12]. An increasing trend with non-B subtypes was thereafter observed with 30% of newly diagnosed cases in 1993 and we predicted that the HIV-1 subtype distribution would become even more diverse [12]. A more recent study confirmed that HIV-1B has decreased over time in patients who were diagnosed between 2003 and 2010 in Sweden [13].

Although the HIV-1 epidemic in Sweden thus shifted from a mono-phylogenetic HIV-1B epidemic to a more diverse pattern over time [12], [13], the detailed changes since the start of the epidemic up to now have not yet been described. We therefore re-subtyped all available sequences from HIV-1 infected individuals diagnosed in Sweden between 1983 and 2012, and described the temporal trends the HIV-1 subtypes and recombinants over three decades.

## Material and Methods

### Patients and dataset

The data were derived from the Swedish InfCare HIV database, which at the 31^st^ December 2012 included >99% of living adult Swedish residents with confirmed HIV infection (n = 6374), and the majority of all patients diagnosed 1983–2012 (n = 9146), at 30 clinics from all regions of the country. The Public Health Agency of Sweden report 10332 individuals with HIV diagnosis during this time period (http://folkhalsomyndigheten.se/about-folkhalso myndigheten-the-public-health-agency-of-sweden/) but in the official statistics the numbers are over reported due to duplicates. Of the 9146 individuals, 7068 (77%) subjects have been exposed to combined antiretroviral therapy (ART) with three drugs or more from 1996, and at 31^st^ of December 2012, 5872 (92.1%) were active on ART. Since 1983, 747 patients have left the country, 61 have been reported missing and 1813 patients have died, as reported in the database.

The database contains demographic data, clinical data, treatment history and the results of almost all routine genotypic resistance testing (GRT) ever performed, including the viral sequences, starting in 1992 [14]. In the national guidelines, routine GRT has been recommended for patients who fail ART and for newly diagnosed patients since 2001 [15]. In 587 out of the 2501 (23%) patients who were diagnosed between 1981 and 1991, the viruses have been sequenced retrospectively. Of the remaining patients from the 1980s, >700 subjects have earlier been serotyped and/or genotyped as belonging to mainly HIV-1B [12]. After excluding 33 HIV-2 patients, 253 patients with no registered date of HIV-1 diagnosis and 4925 patients with no sequence available in the database for re-subtyping, the present dataset included sequences from 3967 HIV-1 infected individuals (Table 1). The year wise HIV-1 diagnosis among the different transmission risk group is presented in table S1. Among the patients without sequences diagnosed before 1992, 49.7% were men who have sex with men (MSM), 21.8% were intra-venous drug users (IVDU), 18.5% were heterosexually infected and 10% were others/unknown. From 1992 up to 2012, the corresponding proportions were MSM: 20%; IVDU: 6.9%; heterosexually infected: 58.2% and others/unknown: 14.9%.

**Table 1:** Demographics of individuals diagnosed with HIV-1 during 1983–2012 in Sweden, included in the study (n = 3967).

		B	C	Subtype Recombinant	Other Pure	All	P-value
**Female**	N (%)	206(11.0)	414(57.0)	380(44.2)	277(54.0)	1277(32.2)	<0.001**
**CD4/µl at diagnosis**	Median (IQR)	410 (243–593)	289 (161–440)	310 (140–510)	330 (180–500)	352 (189–534)	<0.001*
**Year of diagnosis**	Median (IQR)	2000 (1991–2007)	2005 (2000–2009)	2007 (2004–2010)	2005 (1998–2009)	2004 (1996–2008)	<0.001*
**Year of sequence**	Median (IQR)	2003 (1998–2008)	2007 (2003–2010)	2008 (2005–2010)	2007 (2003–2010)	2006 (2001–2009)	<0.001*
Route of transmission***							
**IVDU**	N (%)	218(11.7)	2(0.3)	96(11.2)	11(2.1)	327(8.2)	
**Hetero Male**	N (%)	130(7.0)	227(31.3)	262(30.5)	160(31.2)	779(19.6)	
**Hetero Female**	N (%)	127(6.8)	341(47.0)	314(36.5)	228(44.4)	1010(25.5)	
**MSM**	N (%)	1334(71.4)	21(2.9)	98(11.4)	38(7.4)	1491(37.6)	
**Other**	N (%)	59(3.2)	135(18.6)	90(10.5)	76(14.8)	360(9.1)	
**Total**	N (%)	1868(100)	726(100)	860(100)	513(100)	3967(100)	<0.001**
Country of birth****							
**Sweden**	N (%)	1239(68.2)	82(11.4)	307(36.1)	97(19.1)	1725(44.3)	
**Africa - East and Southern**	N (%)	20(1.1)	459(64.0)	42(4.9)	197(38.8)	718(18.4)	
**Africa - West and Central**	N (%)	9(0.5)	74(10.3)	164(19.3)	65(12.9)	312(8.0)	
**Asia and Pacific**	N (%)	75(4.1)	11(1.5)	218(25.6)	19(3.7)	323(8.3)	
**Caribbean**	N (%)	25(1.4)	0(0)	3(0.4)	1(0.2)	29(0.8)	
**Eastern Europe and Central Asia**	N (%)	69(3.8)	2(0.3)	35(4.1)	51(10.0)	157(4.0)	
**Latin America**	N (%)	119(6.6)	2(0.3)	16(1.9)	11(2.2)	148(3.8)	
**North Africa and Middle East**	N (%)	57(3.1)	75(10.5)	29(3.4)	47(9.2)	208(5.3)	
**Other**	N (%)	204(11.2)	12(1.8)	36(4.2)	20(3.9)	272(7.0)	
**Total**	N (%)	1817(100)	717(100)	850(100)	508(100)	3892(100)	<0.001**
Country of transmission****							
**Sweden**	N (%)	1160(70.4)	73(11.0)	236(29.2)	82(17.6)	1551(43.3)	
**Africa - East and Southern**	N (%)	21(1.3)	408(61.6)	38(4.7)	181(38.8)	648(18.1)	
**Africa - West and Central**	N (%)	1(0.06)	67(10.1)	156(19.3)	62(13.3)	286(8.0)	
**Asia and Pacific**	N (%)	80(4.9)	13(2.0)	308(38.2)	22(4.7)	423(11.8)	
**Caribbean**	N (%)	6(0.4)	0(0)	2(0.2)	0(0)	8(0.2)	
**Eastern Europe and Central Asia**	N (%)	21(1.3)	1(0.2)	18(2.2)	46(9.9)	86(2.4)	
**Latin America**	N (%)	62(3.8)	1(0.2)	8(1.0)	4(0.9)	75(2.1)	
**North Africa and Middle East**	N (%)	24(1.5)	75(11.3)	16(2.0)	34(7.3)	149(4.2)	
**Other**	N (%)	272(16.5)	24(3.6)	25(3.1)	35(7.5)	356(9.9)	
**Total**	N (%)	1647(100)	662(100)	807(100)	466(100)	3582(100)	<0.001**

*Kruskal-Wallis equality-of-populations rank test; **Chi2 test: ***Intravenous drug users: IVDU; Hetero: heterosexually infected; MSM: men who have sex with men; Others: blood products or unknown; ****The countries were divided into categories based on the UNAIDS definition (http://www.unaids.org/en/regionscountries/countries/).

### Sequences and subtyping

For accurate identification of recombinant forms only partial *pol* sequences that had a minimum length of 500 nucleotides (nt) were used. For patients with multiple sequences (1253/3967; 32%), the oldest sequence was used for subtyping and the most recent sequence was used to verify the subtype.

HIV-1 subtyping was inferred initially by two methods: i) REGA subtyping tool version 3 (REGA v3), which uses an improved decision-tree algorithm and has greater sensitivity for identification of pure subtypes and recombinants [11]; and ii) COMET (http://comet.retrovirology.lu/), which uses context-based modelling for expeditious typing of HIV-1 viruses. Both the tools showed best performances for pure subtypes and CRFs [11]. Additionally we used Recombinant identification program version 3 (RIP 3.0) with 200 nt window size, which determines recombination in shorter sequences (<700 nt) [http://www.hiv.lanl.gov/content/sequence/RIP/RIPexplain.html]; and HIV-1 BLAST, available in the HIV Los Alamos Database [http://www.hiv.lanl.gov/content/sequence/BASIC_BLAST/basic_blast.html], to identify the nearest HIV-1 sequences from different geographical proximity. Due to limitations such as differences in the length of the sequences, the presence of degenerate bases “N” inside the sequences (especially older sequences), thus detailed phylogenetic analysis was not done. Further, the presence of drug resistance mutations can affect the phylogenetic inference but not affect subtyping and the length of the sequences could be adjusted in the alignment in the automated tools. However, the REGA v3 uses phylogenetic methods to identify the subtype of a specific sequence.

A sequence was designated as a particular subtype if two of the three tools (REGA, COMET or RIP) determined it. The sequences were designated as pure subtype [A (A1 and A2), B, C, D, F (F1 and F2), G, H, J, and K], circulating recombinant forms (CRFs) [CRF01 to CRF49 as of May 2013], and unique recombinant forms (URFs). The URFs were further characterised using bootscan analysis incorporated in the Rega v3 tool and validated in the SimPlot v3 software [16].

### Statistical analysis

Demography and clinical parameters were assessed by Kruskal-Wallis equality-of-populations rank test for continuous variables and χ^2^ test for categorical variables. To assess trends of the HIV-1 subtypes, a multinomial logistic regression model [17] was used for the 3967 patients with HIV diagnosis between 1983 and 2012. The outcome (the HIV-1 subtypes) was grouped into HIV-1B (reference group), HIV-1C, recombinants, and other pure subtypes. The year of HIV-1 diagnosis was the only covariate considered within the model. Square root of year of diagnosis was also added in the model to test for non-linearity, but was not significant. As sensitivity analysis and to get a more detailed description of which strains were primarily imported to Sweden and which strains actually have spread in Sweden, we performed analyses restricted to: 1) All patients infected in Sweden (n = 1551); 2) Patients heterosexually infected in Sweden (n = 307); 3) MSM infected in Sweden (n = 921); 4) All patients born and infected in Sweden (n = 1165); 5) All patients infected outside of Sweden (n = 2161); 6) All patients born in Sweden and infected outside of Sweden (n = 455); 7) Patients born and infected outside of Sweden (n = 1699). Statistical analyses were performed using Stata version 12.1 SE (StataCorp LP, USA)

### Ethical considerations

The study was approved by Regional Ethics Committée Stockholm (2005/1167–31/3). The patient information was anonymised and de-identified prior to analysis. The authors confirm that there are some restrictions on the data underlying the conclusions in the manuscript. Anonymized sequences may be accessed via Genbank (accession numbers, JQ698667–JQ698874) [13]. The sequences that were analysed are representative of the entire country thereby, in principle, allow for the reconstruction of the transmission network. The entire dataset is available upon request from the steering committee of InfCare HIV.

## Results

### Patient dataset

Patient's demographics are presented in Table 1. Self-reported country of infection was available for 91% (3600/3967) of the patients. Among them, 43% (1551/3600) of the transmission events had occurred in Sweden. The median year of diagnosis differed between the subtypes (HIV-1B: 2000; HIV-1C: 2005; other pure subtypes: 2005; recombinants: 2007) (p<0.001). Compared to HIV-1B, non-B subtypes had a lower median CD4+ T-cell count at diagnosis (B: 410/µl; C: 289/µl; other pure: 310/µl; recombinants: 330/µl) (p<0.001). Also, patients with non-B subtypes were more frequently late presenters than patients with HIV-1B (<200 cells/mm^3^: 27–34% versus 20%; <350 cells/mm^3^: 53–62% versus 40%).

### Diversified presence of HIV-1 subtypes

**Figure 1:**
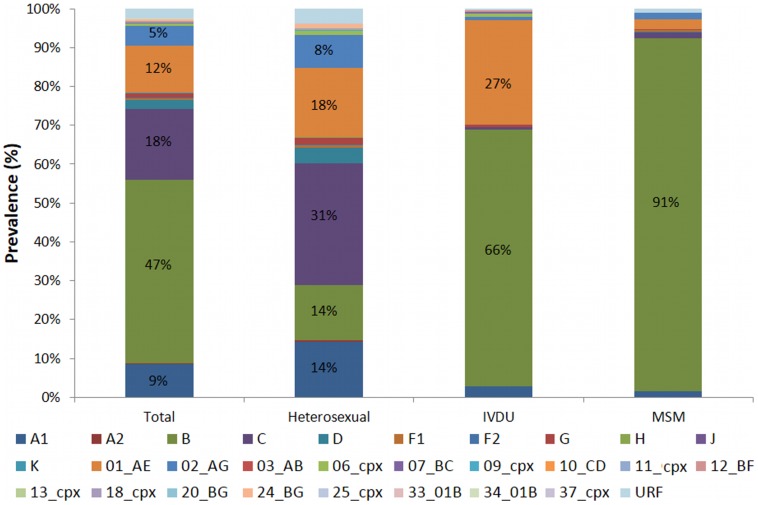
Distribution of HIV-1 subtypes in Sweden between 1983 and 2012. Final dataset included *pol* sequences (length >500 nt) from 3967 HIV-1 infected patients. HIV-1 subtyping was determined by three methods (see method section). The sequences were designated as pure subtypes [A (A1 and A2), B, C, D, F (F1 and F2), G, H, J, and K], circulating recombinant forms (01_AE, 02_AG, 03_AB, 06_cpx, 10_CD, 11_cpx, 12_BF, 13_cpx, 18_cpx, 20_BG, 24_BG, 25_cpx, 33_01B, 34_01B, 37_cpx) and unique recombinant forms (URFs).

All eleven pure subtypes and sub-subtypes within HIV-1 group M (3107/3967; 78%), 17 CRFs (757/3967; 19%), and 32 URFs (103/3967; 3%) were identified (Figure 1). HIV-1B dominated (47%) followed by HIV-1C (18%) and CRF01_AE (12%). HIV-1B dominated in MSM (91%) and IVDU (66%). Among IVDU, 27% were infected with CRF01_AE. A diverse pattern was seen in heterosexually infected patients among whom HIV-1C dominated (31%). The city wise major pure subtypes HIV-1B and HIV-1C along with major CRFs 01_AE and 02_AG was presented in table S2.

### Subtype predictions using different tools

The REGA v3 and COMET showed 96% concordance to determine the subtypes and recombinants forms. The majority of the discordance was observed between RIP 3.0 versus REGA v3 and BLAST on subtyping of CRFs (mainly 01_AE and 02_AG). REGA v3 improved thus the identification of 02_AG compared to the RIP 3.0 tool, as reported earlier [11]. The highest concordance was observed between REGA v3 and BLAST (97%). For the patients for whom there was more than one sequence, a 98% (1207/1230) concordance was observed. The 2% discordance was due to read-length variations, mainly in the terminal region, which limited the possibility to identify recombinants. The median length of the sequences were 1058 nt (range: 551–1758 nt).

### Temporal trends of HIV-1 subtypes and recombinants

**Figure 2:**
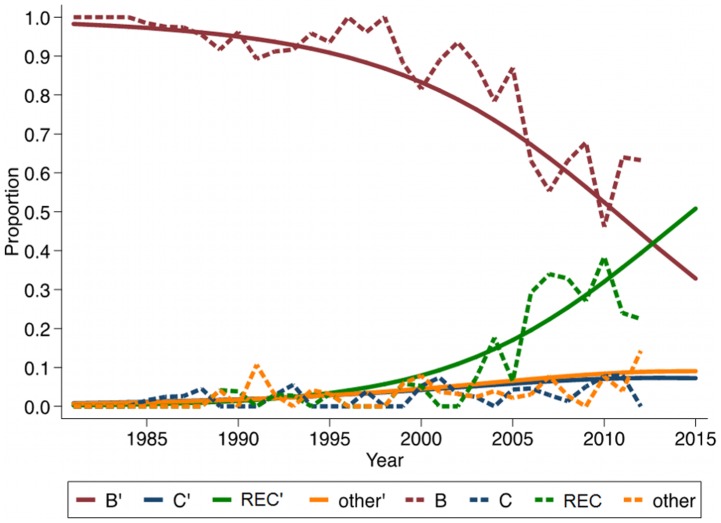
Proportions and predicted proportions of newly diagnosed HIV-1 subtype B (B), subtype C (C), recombinant forms (REC) and other subtypes (other) by year of diagnosis. Dotted lines represent actual prevalence in each year, while the smooth lines represent the predicted prevalence. Multinomial logistic regression model adjusted for year of diagnosis was used.

There was a significant increase of newly diagnosed HIV-1C, recombinants, and other pure subtypes over time compared to HIV-1B (set as reference) (p<0.01) (Figure 2). The multinomial regression model was adjusted for year of diagnosis tested for non-linearity however it was not significant. By extending the graph, the model predicts the exceed of recombinant forms by 2015. In the sensitivity analysis of year of HIV-1 diagnosis, an increase of HIV-1C, recombinant forms and other pure subtypes, using HIV-1B as a reference, was seen for all patients (n = 1551) who were reported to be infected in Sweden. The relative risk ratio (RRR) for HIV-1C was 1.10 (95%CI: 1.06–1.14), for recombinants 1.20 (95%CI: 1.17–1.24) and for other pure subtypes 1.11 (95%CI: 1.07–1.16) (Table 2). The same pattern was seen for patients born and infected in Sweden (n = 1165) (HIV-1C: 1.06, 1.02–1.11; recombinants: 1.22, 1.17–1.27; other pure subtypes: 1.10, 1.05–1.17) and for patients heterosexually infected in Sweden (n = 307) (HIV-1C: 1.12, 1.06–1.18; recombinants: 1.19, 1.13–1.27; other pure subtypes: 1.14, 1.08–1.20). For MSM patients infected in Sweden (n = 921), recombinant forms (1.27, 0.99–1.19) and other pure subtypes (1.1, 1.05–1.17) increased significantly compared to HIV-1B, but not HIV-1C (1.08, 0.99–1.19).

**Table 2:** Patients infected in Sweden - sensitivity analysis of year of HIV-1 diagnosis and the trends in subtype pattern during 1983–2012, using subtype B (HIV-1B) as reference.

	All patients infected in Sweden		Patients born and infected in Sweden		MSM infected in Sweden		Heterosexually infected in Sweden	
	(n = 1551)		(n = 1165)		(n = 921)		(n = 307)	
	RRR (95% CI)	p-value	RRR (95% CI)	p-value	RRR (95% CI)	p-value	RRR (95% CI)	p-value
								
**HIV-1B**	Ref.		Ref.		Ref.		Ref.	
**HIV-1C**								
Year of diagnosis	1.10 (1.06–1.14)	<0.001	1.06 (1.02–1.11)	0.007	1.08 (0.99–1.19)	0.096	1.12 (1.06–1.18)	<0.001
**Recombinants**								
Year of diagnosis	1.20 (1.17–1.24)	<0.001	1.22 (1.17–1.27)	<0.001	1.27 (1.17–1.37)	<0.001	1.19 (1.13–1.27)	<0.001
**Other**								
Year of diagnosis	1.11 (1.07–1.16)	<0.001	1.10 (1.05–1.17)	<0.001	1.22 (1.07–1.38)	0.003	1.14 (1.08–1.20)	<0.001

An multinomial logistic regression model [17] was used. The outcome (the HIV-1 subtypes) was grouped into HIV-1B (reference group), HIV-1C, recombinants and other pure subtypes. Relative risk ratio and 95% confidence intervals were determined. RRR represents relative risk ratio.

In the second sensitivity analysis of the year of diagnosis restricted to the patients infected outside Sweden (Table 3), a significant increase of HIV-1C (1.07, 1.06–1.09), recombinant forms (1.15, 1.12–1.17) and other pure subtypes was seen (1.05; 1.04–1.07) when all patients (n = 2161) were analysed as well as for patients (n = 1699) who were born and infected outside Sweden (HIV-1C: 1.08, 1.05–1.11; recombinant forms: 1.14, 1.11–1.17; other pure subtypes: 1.05, 1.03–1.07). For patients (n = 455) born in Sweden but infected outside, recombinants (1.16, 1.11–1.20) and other pure subtypes (1.08, 1.03–1.13) were significantly increased, but not HIV-1C (1.03, 0.02–1.07).

**Table 3:** Patients infected outside of Sweden - sensitivity analysis of year of HIV-1 diagnosis and the trends in subtype pattern during 1983–2012, using subtype B (HIV-1B) as reference.

	All patients infected outside Sweden		Patients born in Sweden and infected outside Sweden		Patients born and infected outside Sweden	
	(n = 2161)		(n = 455)		(n = 1699)	
	RRR (95% CI)	p-value	RRR (95% CI)	p-value	RRR (95% CI)	p-value
**HIV-1B**	Ref.		Ref.		Ref.	
**HIV-1C**						
Year of diagnosis	1.07 (1.06–1.09)	<0.001	1.03 (0.02–1.07)	0.216	1.08 (1.05–1.1)	<0.001
						
**Recombinants**						
Year of diagnosis	1.15 (1.12–1.17)	<0.001	1.16 (1.11–1.20)	<0.001	1.14 (1.11–1.17)	<0.001
						
**Other**						
Year of diagnosis	1.05 (1.04–1.07)	<0.001	1.08 (1.03–1.13)	<0.001	1.05 (1.03–1.07)	<0.001
						

An univariate multinomial logistic regression model [17] was used. The outcome (the HIV-1 subtypes) was grouped into HIV-1B (reference group), HIV-1C, recombinants, and other pure subtypes. Relative risk ratio and 95% confidence intervals were determined. RRR represents relative risk ratio.

### Mosaic pattern of URFs

**Figure 3:**
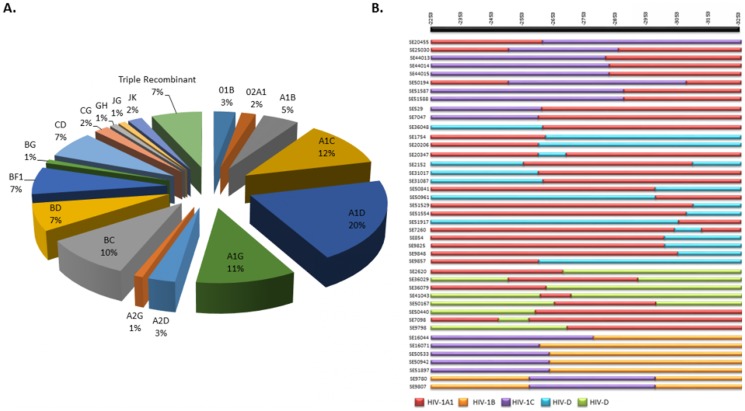
Types and mosaic pattern of HIV-1 unique recombinant forms identified in Sweden. **A**. Proportion of recombinant forms was identified based on REGAv3, COMET and RIP 3.0 followed by bootscan analysis incorporated in REGAv3 and SimPlot analysis. **B. Mosaic pattern of URFs identified by REGAv3 and SimPlot.** Major URFs (A1C, A1D, BC, A1G) which have HXB2 positions 2253 to 3260 and had minimum degenerate bases (≤5) were used for bootscan analysis (n = 43) implemented in Rega v3 with 400 bp window size and 20 bp step size and SimPlot analysis with 300 bp window size and 20 bp step size. The reference sequences were selected for SimPlot analysis is based on the recombinant pattern. Upper bar presents HXB2 co-ordinate.

A total of 103 patients (3%) were infected by URFs. Recombinants between HIV-1A1 and D (A1D) were the most common (n = 21), followed by A1C (n = 12), A1G (n = 11), BC (n = 10), BF1, BD, CD, and complex triple recombinants (n = 7, each) (Figure 3A). Further major URFs (A1C, A1D, BC, A1G) which have HXB2 positions 2253 to 3260 and had minimum degenerate bases (≤5) were used for bootscan analysis (n = 43) implemented in Rega v3 with 400 bp window size and 20 bp step size and SimPlot analysis with 300 bp window size and 20 bp step size. The breakpoint analysis showed 33 different mosaic patterns of recombinants (Figure 3B), indicating a presence of diverse recombinants in the country.

### Movement of major HIV-1 subtypes and recombinant forms

Based on the self-reported country of transmission along with the migration history in the country and probable geographic linkages of the sequences using BLAST analysis, the predicted movements of the HIV-1 strains were described. HIV-1C entered from three different regions: East Africa, Southern Africa and West-Central Africa (Burundi and Congo). CRF01_AE migrated from mainly Thailand and CRF02_AG from West-Central Africa. A fraction of 06_cpx migrated from Eastern Europe - Central Asia. HIV-1B was mainly introduced from Latin America, United States and Western Europe, although a small fraction was introduced from Thailand.

## Discussion

The Swedish HIV-1 epidemic started in December 1979 in Stockholm with an outbreak of HIV-1B infection among MSM [18]. We describe now the changes of the diversity of the HIV-1 subtypes during three decades, using viral sequences from nearly 4000 patients, representing almost half of all diagnosed patients in Sweden ever. Our analysis shows that all eleven group M pure subtypes and sub-subtypes, 17 CRFs and several URFs have been introduced into the country and that there is a trend of increasing new diagnosis of HIV-1C and CRFs, as compared to HIV-1B.

The initial spread of HIV-1 in Western Europe was driven by HIV-1B [19]. Although, HIV-1B was still predominant among the MSM and the IVDUs in the present study, as also observed in a cross-sectional multinational European study [20], the prediction of a further diversification of HIV-1 subtypes in Sweden due migration was confirmed [12]. When virus sequences from all patients were analysed, increasing trends of HIV-1C, recombinants (CRFs and URFs) and other pure subtypes were thus observed. This strong increase of viral diversity at the national level was to a large extent due to human mobility from high prevalence countries, both from those where such a viral heterogeneity pattern prevails and from countries where the epidemics are largely mono-phylogenetic, such as Ethiopia and Thailand.

In addition to the migration of already HIV-1 infected individuals, we found that patients who were born and infected in Sweden contributed to the changing HIV-1 subtypes as well as those born abroad but reported to be infected in Sweden, as suggested by our sensitivity analysis. This was true also for MSM infected in Sweden where an increase of CRFs and other pure subtypes was described. Thus, the changing Swedish HIV-1 subtype pattern is not only due to migration of persons who were infected at arrival, but also to a spread of non-subtype B strains among migrants after arrival to the country and MSM. It shall be noted that the country of infection was self-reported and data collected this way are likely to underestimate the proportion of migrants infected after arrival to a country [21]. It is therefore not unlikely that the infection with non-B subtypes among persons living in Sweden is even higher than reported in the present study. Our finding of an increase of HIV-1C infections among those reported to have become infected in Sweden is line with the reports that in other regions where HIV-1C has been introduced, its prevalence has increased gradually. E.g. in Brazil, a temporal increase in the prevalence of HIV-1C and related recombinants has been observed over time [22], [23].

A contribution to the relative increase in non-HIV-1B infections among newly diagnosed patients in Sweden could be that undiagnosed HIV-1 infection is more common among migrants, as compared to MSM [24]. The time of diagnosis is shifted away from time of infection towards more recent years. Thus, the T-cell counts at diagnosis were lower among non-B infected patients and late presentation was more common compared to HIV-1B infected subjects. Also, differences in the coverage rate and efficacy of ART may lead to a relative decrease in the number of persons infected by HIV-1B strains within the country since undetectable viremia due to ART is associated with minimal contagiousness [25]. Thus, although the general coverage rate in Sweden is presently high, >90%, the proportion of successfully treated MSM is even higher than among heterosexually infected individuals [24]. Considering the actual change in proportions over calendar years and the analysis predictions, the recombinant forms will probably dominate by the year 2015 among newly diagnosed patients in Sweden. But it is obvious that the development will still be highly dependent on the migration patterns for the coming years.

There are some caveats that need to be mentioned. Half of the patients had not sequences available for subtyping, among whom MSM dominated among those diagnosed before 1992 and migrants among those diagnosed later. However, there was no difference in gender and mode of transmission between those having sequences and those who did not have sequences. Also, most of these patients were diagnosed during the 1980s and we have earlier shown that the vast majority of patients during this time period were infected by HIV-1B [12]. At least 491 patients entered into the cohort when they were on therapy and due to non-detectable viral load, genotyping was not performed. However, the majority of these patients were migrants from Africa. Altogether, the trend in increase of non-HIV-1B is unlikely to be over-estimated, rather the opposite. Another caveat is that the country of infection was assessed through self-reports, but this bias would also rather result in an underestimation of non-HIV-1B [21]. It shall however be noted that it is not uncommon the migrants visit their home country and that they might become infected there [21]. Thus, our study cannot precisely define the actual spread of non-B strains within Sweden. It is also likely that the prevalence of recombinant forms was underestimated since only the *pol* region was sequenced, as earlier described for the Indian HIV-1 epidemic when only one gene was analysed [3]. Despite these caveats, we believe that the strength of the study is that it covers data from the beginning of HIV epidemic till date and includes almost all sequences performed in routine diagnostics in Sweden, corresponding to almost 4000 individuals.

In conclusion, this is the first comprehensive study to describe the trends of the HIV-1 subtype distribution in Sweden since the beginning of the pandemic. To best of our knowledge, the epidemic is one of the most diverse epidemics outside west-central Africa with presence of all pure HIV-1 M group subtype, 17 CRFs and 32 URFs. Countries such as Portugal, Belgium, Switzerland and more recently, UK and Germany are having similar trends with an increasing of non-B subtypes [20], [26], [27]. Presently, there seems to be no or limited transmission of HIV-1 between the major populations at risk but if sexual intermingling increases in the future, the viral heterogeneity may become a hotspot for diverse and complex URFs. Such a scenario is supported by the spreading of novel recombinants across multiple risk groups in the United Kingdom [28] and that several triple URFs already are circulating in Sweden. Apart from that HIV-1B was also found to be Continual molecular surveillance is recommended to understand the changing nature of the epidemic and the mobility of the HIV-1 strains.

## Supporting Information

Table S1 **The Table describes the number of newly diagnosed patients whose virus was sequenced each year from 1983 to 2012, both in total and for the different categories of patients.** IVDU: intravenous drug users; MSM: men who have sex with men; Others: infected through blood products and unknown.
(DOCX)

Table S2 **The table describes the city wise distribution of HIV-1 major subtypes and recombinants (HIV-1B, HIV-1C, 01_AE and 02_AG) analysed in this study.**
(DOCX)
