# Supplementary material for: Temporal Trends in the Swedish HIV-1 Epidemic: Increase in Non-B Subtypes and Recombinant Forms over Three Decades
Source: PLoS One. 2014 Jun 12;9(6):e99390. doi: 10.1371/journal.pone.0099390 (PMC4055746; doi:10.1371/journal.pone.0099390)
Supplement: Table S1 — The Table describes the number of newly diagnosed patients whose virus was sequenced each year from 1983 to 2012, both in total and for the different categories of patients. IVDU: intravenous drug users; MSM: men who have sex with men; Others: infected through blood products and unknown. (DOCX) [file pone.0099390.s001.docx]

**Table S1.** The Table describes the number of newly diagnosed patients whose virus is sequenced each year from 1983 to 2012, both in total and for the different categories of patients. IVDU: intravenous drug users; MSM: men who have sex with men; Others: infected through blood products and unknown.

| Year of HIV diagnosis | *IDVU* | *Heterosexual* | *MSM* | *Other* |
| --- | --- | --- | --- | --- |
|  | N (%) | N (%) | N (%) | N (%) |
| 1983 | 1(0.31) | 1(0.06) | 8(0.54) | 2(0.56) |
| 1984 | 7(2.14) | 1(0.06) | 28(1.88) | 3(0.83) |
| 1985 | 30(9.17) | 3(0.17) | 64(4.29) | 7(1.94) |
| 1986 | 16(4.89) | 7(0.39) | 44(2.95) | 5(1.39) |
| 1987 | 16(4.89) | 21(1.17) | 38(2.55) | 3(0.83) |
| 1988 | 5(1.53) | 11(0.61) | 31(2.08) | 2(0.56) |
| 1989 | 11(3.36) | 21(1.17) | 22(1.48) | 8(2.22) |
| 1990 | 10(3.06) | 39(2.18) | 24(1.61) | 7(1.94) |
| 1991 | 7(2.14) | 41(2.29) | 39(2.62) | 4(1.11) |
| 1992 | 10(3.06) | 41(2.29) | 44(2.95) | 9(2.5) |
| 1993 | 5(1.53) | 48(2.68) | 54(3.62) | 8(2.22) |
| 1994 | 7(2.14) | 23(1.29) | 38(2.55) | 5(1.39) |
| 1995 | 4(1.22) | 42(2.35) | 42(2.82) | 7(1.94) |
| 1996 | 5(1.53) | 32(1.79) | 40(2.68) | 5(1.39) |
| 1997 | 4(1.22) | 39(2.18) | 38(2.55) | 4(1.11) |
| 1998 | 3(0.92) | 31(1.73) | 29(1.95) | 3(0.83) |
| 1999 | 6(1.83) | 35(1.96) | 22(1.48) | 8(2.22) |
| 2000 | 5(1.53) | 53(2.96) | 53(3.55) | 10(2.78) |
| 2001 | 5(1.53) | 51(2.85) | 29(1.95) | 12(3.33) |
| 2002 | 9(2.75) | 59(3.3) | 42(2.82) | 15(4.17) |
| 2003 | 11(3.36) | 101(5.65) | 46(3.09) | 17(4.72) |
| 2004 | 12(3.67) | 132(7.38) | 60(4.02) | 14(3.89) |
| 2005 | 13(3.98) | 98(5.48) | 78(5.23) | 16(4.44) |
| 2006 | 25(7.65) | 119(6.65) | 78(5.23) | 21(5.83) |
| 2007 | 46(14.07) | 119(6.65) | 88(5.9) | 26(7.22) |
| 2008 | 17(5.2) | 133(7.43) | 99(6.64) | 29(8.06) |
| 2009 | 15(4.59) | 134(7.49) | 106(7.11) | 29(8.06) |
| 2010 | 11(3.36) | 107(5.98) | 46(3.09) | 33(9.17) |
| 2011 | 8(2.45) | 142(7.94) | 78(5.23) | 30(8.33) |
| 2012 | 3(0.92) | 105(5.87) | 83(5.57) | 18(5) |
| **Total** | **327(100)** | **1789(100)** | **1491(100)** | **360(100)** |
