# Supplementary material for: Temporal Trends in the Swedish HIV-1 Epidemic: Increase in Non-B Subtypes and Recombinant Forms over Three Decades
Source: PLoS One. 2014 Jun 12;9(6):e99390. doi: 10.1371/journal.pone.0099390 (PMC4055746; doi:10.1371/journal.pone.0099390)
Supplement: Table S2 — The table describes the city wise distribution of HIV-1 major subtypes and recombinants (HIV-1B, HIV-1C, 01_AE and 02_AG) analysed in this study. (DOCX) [file pone.0099390.s002.docx]

**Table S2.** City wise distribution of HIV-1 major subtype and CRFs in Sweden

| City | Total | Major Subtypes; N (%) | | | | |
| --- | --- | --- | --- | --- | --- | --- |
|  |  | B | C | 01_AE | 02_AG | Other |
| Huddinge | 1072 | 434 (40) | 181 (17) | 166 (15) | 72 (7) | 219 (20) |
| Sahlgrenska/Ostra | 263 | 91 (35) | 23 (3) | 29 (11) | 34 (13) | 40 (15) |
| Venhalsan | 911 | 779 (860 | 23 (3) | 24 (3) | 12 (1) | 73 (8) |
| Solna | 486 | 223 (46) | 117 (24) | 46 (9) | 12 (2) | 88 (18) |
| Uppsala | 70 | 22 (31) | 17 (24) | 13 (19) | 1 (1) | 17 (24) |
| Vasteras | 38 | 7 (18) | 18 (47) | 5 (13) | 3 (8) | 5 (13) |
| Eskilstuna | 47 | 10 (21) | 13 (32) | 8 (17) | 1 (2) | 15 (32) |
| Sunderbyn | 75 | 9 (12) | 32 (43) | 10 (13) | 3 (4) | 21 (28) |
| Falun | 36 | 3 (8) | 11(31) | 12 (33) | 0 | 10 (28) |
| Boras | 28 | 9 (32) | 9 (32) | 4 (14) | 2 (7) | 4 (14) |
| Skovde | 24 | 5 (21) | 5 (21) | 5 (21) | 3 (13) | 6 (25) |
| Jonkoping | 28 | 9 (32) | 2 (7) | 5 (18) | 5 (18) | 7 (25) |
| Sahlgrenska Hud & Konssjukvard | 49 | 27 (55) | 4 (8) | 6 (12) | 3 (6) | 9 (18) |
| Gavle | 101 | 14 (14) | 41 (41) | 25 (25) | 5 (5) | 16 (16) |
| Orebro | 46 | 9 (20) | 20 (43) | 6 (13) | 2 (4) | 9 (20) |
| Visby | 1 | 0 | 0 | 0 | 0 | 1 (100) |
| Vaxsjo | 35 | 4 (11) | 8 (23) | 5 (14) | 1 (3) | 17 (49) |
| Lund | 56 | 19 (34) | 11 (20) | 11 (20) | 3 (5) | 12 (21) |
